# Interactive breastfeeding theory: fawcettʼs pragmatic adequacy assessment

**DOI:** 10.1590/1980-220X-REEUSP-2024-0144en

**Published:** 2024-12-02

**Authors:** Samara Calixto Gomes, Icleia Parente Rodrigues, Marcos Venícius de Oliveira Lopes, Viviane Martins da Silva, Nirla Gomes Guedes, Patricia Neyva da Costa Pinheiro

**Affiliations:** 1 Universidade Federal do Ceará, Departamento de Enfermagem, Fortaleza, CE, Brazil.

**Keywords:** Nursing Theory, Evaluation study, Maternal and child health, Breast feeding, Teoría de enfermería, Estudio de evaluación, Salud Materno-Infantil, Lactancia materna

## Abstract

**Objective:**

To assess the pragmatic adequacy of the Interactive Theory of Breastfeeding based on the Fawcett’s Model.

**Method:**

Theoretical study, according to evaluation criteria proposed by Fawcett. The six questions suggested in the model were used, answered based on searches in scientific literature, consultation in legislation supporting nursing professional practice and information from the authors of the theory.

**Results:**

It was observed that during their training, Nurses acquire skills for care, but clinical management focused on breastfeeding should be emphasized. Since its creation, the theory has been applied in an ascending manner, proving its application in clinical practice to be viable. The existence of laws and resolutions guarantee clinical nurses the legal support necessary to develop a practice based on the Interactive Theory of Breastfeeding.

**Conclusion:**

The pragmatic adequacy of the theory was observed, showing that the Interactive Theory of Breastfeeding subsidizes a care directed at the mother and child binomial, supporting the nurse in their decision-making, contributing to the protection, promotion, and support of breastfeeding.

## RESUMEN

**Objetivo:**

Realizar una evaluación de la adecuación pragmática de la Teoría de la Lactancia Materna Interactiva basada en el Modelo de Fawcett.

**Método:**

Estudio teórico, según criterios de evaluación propuestos por Fawcett. Se utilizaron las seis preguntas sugeridas en el modelo, respondidas a partir de una búsqueda en la literatura científica, consulta de la legislación que sustenta el ejercicio profesional de la enfermería e información de los autores de la teoría.

**Resultados:**

Se observó que durante su formación el enfermero adquiere habilidades de cuidado, pero es necesario intensificar la gestión clínica enfocada a la lactancia materna. Desde su creación, la teoría se ha aplicado de forma ascendente, demostrando que su aplicación en la práctica clínica es viable. La existencia de leyes y resoluciones garantizan a las enfermeras clínicas el respaldo legal necesario para el desarrollo de una práctica sustentada en la Teoría de la Lactancia Materna Interactiva.

**Conclusión:**

Se observó la adecuación pragmática de la teoría, demostrando que la Teoría de la Lactancia Materna Interactiva subvenciona atención dirigida al binomio madre e hijo, apoyando al enfermero en su toma de decisiones, contribuyendo a la protección, promoción y apoyo a la lactancia materna.

## INTRODUCTION

A study carried out on breastfeeding in 153 countries highlights Brazil as a world reference, recording an adherence rate of 41%^(1)^. However, there is still a long way to go to reach the 70% target set by the World Health Organization (WHO) and the United Nations Children’s Fund (UNICEF) by 2030^(2)^.

The act of breastfeeding is much more than simply offering human milk. It is a multifactorial and complex practice that can be directly influenced by some aspects, such as: the perception and biological conditions of the mother and child binomial, space for breastfeeding, the mother’s role and her decision-making^(3)^.

In the complexity surrounding breastfeeding, the Interactive Theory of Breastfeeding stands out, describing it as a systemic, dynamic, and procedural dimension. It is considered “a process of dynamic interaction in which mother and child interact with each other and with the environment to achieve the benefits of human milk provided directly from the breast to the child, being a unique experience at each event”^(3,4)^. This theory presents as a theoretical framework the Conceptual Model of Open Systems by Imogene King^(5)^.

In the context of nursing care, theories contribute as a theoretical framework for a symbolic representation of aspects of reality, legitimizing an occupation based on its ability to create and apply theories. In practice, they promote knowledge as a guide for nurses’ actions^(6)^.

Theories present elements capable of describing, explaining, predicting, or prescribing conditions or relationships among phenomena^(5,7)^. By applying them, nurses promote a systematic means for carrying out critical reflection, assisting the nurse’s practice based on theoretical references^(8)^.

A theory generally arises from the need to express a new idea or phenomenon, being an important link between knowledge and practice^(6)^. The use of theories structures and organizes nursing knowledge, providing autonomy in care, bringing important reflections for the visibility of nursing as a science^(8)^.

Thus, it is worth highlighting the importance of evaluating a theory and its components to improve the development of nursing practice, for it to be able to direct the structuring of research, teaching, administration and nursing consultation, making changes in clinical practice, and encouraging nurses to be critical consumers of theories and evidence-based practice^(7)^.

To be evaluated, a theory requires that judgments be made about the extent to which it meets certain criteria. Therefore, Fawcett^(9)^ determines, as one of her criteria for evaluating a theory, Pragmatic Adequacy, which refers to the usefulness of a theory in nursing practice and the measurement of outcomes in terms of effectiveness in problem-solving.

Thus, this study aims to assess the pragmatic adequacy of the Interactive Theory of Breastfeeding based on Fawcett’s Model. The study of this theory is justified since it aims in-depth understanding and dissemination of its concepts for the nursing clinical practice.

## METHOD

Theoretical study to evaluate the practical applicability of the Interactive Theory of Breastfeeding, using the evaluation criteria proposed by Jacqueline Fawcett^(9)^, which involve judgments related to the reliability of a theory, defined in six criteria: significance, internal consistency, parsimony, testability, empirical adequacy and, lastly, pragmatic adequacy^(10)^.

Pragmatic adequacy is the sixth criterion for evaluating a theory and is based on its practical applicability, requiring nurses to understand its content and the skills necessary for its application, so that they can achieve favorable results from care actions^(9)^. This approach emphasizes the effectiveness of a theory in solving problems and seeks to determine whether what is intended or experienced achieves its purpose^(10)^.

This criterion requires that, through significant actions, the theory promotes positive results, such as: reduction of complications, improvement in health conditions and increased satisfaction of all participants^(10)^.

**Figure 1:**
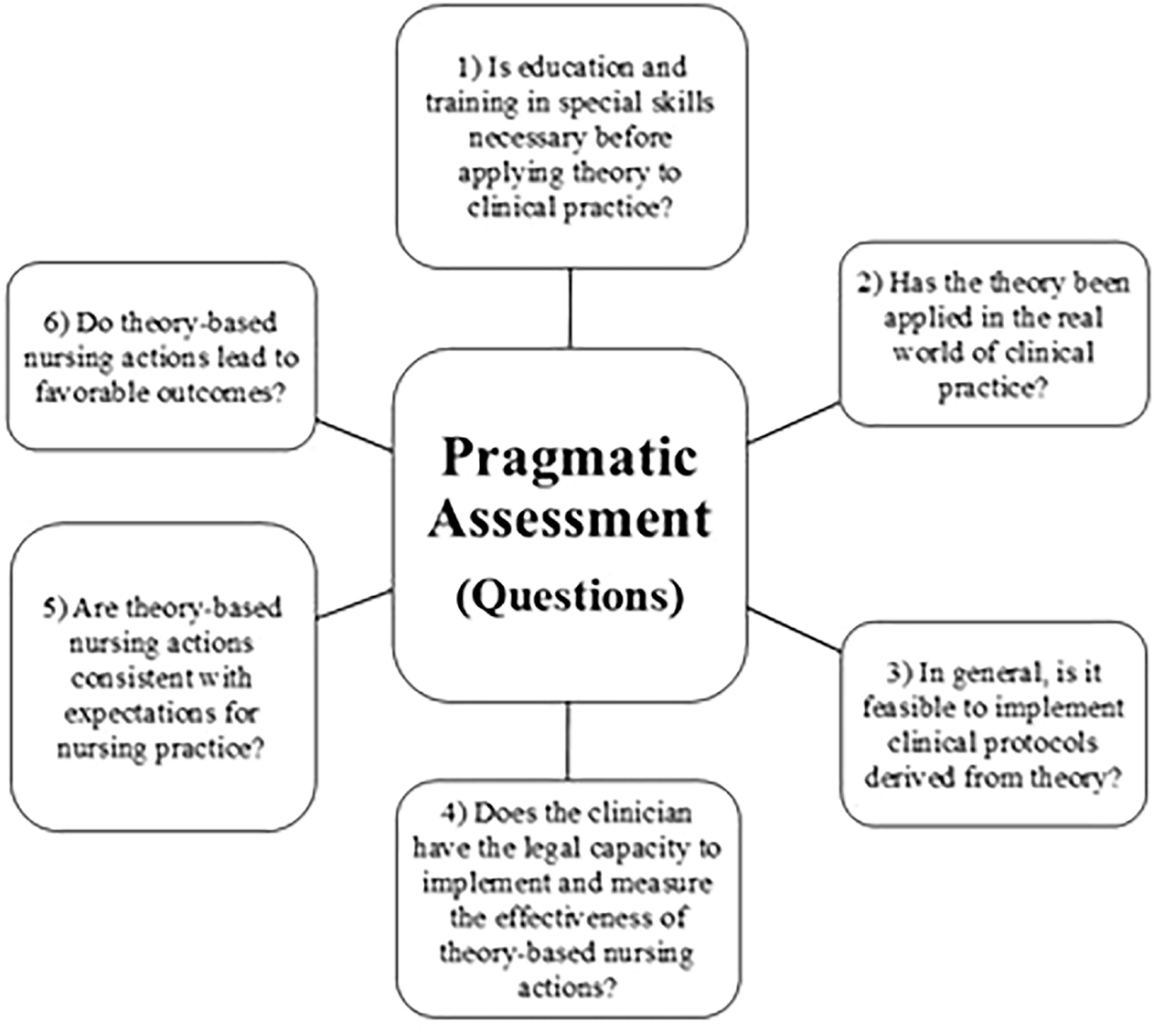
Questions proposed by Fawcett for pragmatic evaluation.

Source: Authors’ production

To assess the pragmatic adequacy of the Interactive Theory of Breastfeeding, the questions suggested by Fawcett, presented in Figure 1, were applied.

The first question investigates whether there is a need for specific training to apply the theory in clinical practice. Initially, the term competencies stands out as the “ability to articulate values, knowledge, skills, and attitudes necessary for the effective performance of activities required by the nature of the work and the achievement of the established objectives”^(11)^. In this regard, the terms *Skills* and *Soft Skills* should be recognized, where the former refers to technical skills, which can be validated directly, through objective tests, or indirectly, by presenting a certificate of completion of a course, and the latter refers to socio-emotional or non-technical skills, such as communication skills, teamwork skills and leadership^(11)^.

To map the answers to the second, third, and sixth questions, a scoping review was carried out based on the JBI recommendations^(12)^, following these steps: definition and alignment of objectives and guiding question; development of inclusion and exclusion criteria; description of the search planning, selection, data extraction, and presentation of outcomes.

The objective of the review was to map the contributions of the Interactive Theory of Breastfeeding to clinical nursing practice. The research question was formulated based on the PCC strategy, which corresponds to the acronym *Population, Concept, Context* (PCC)^(12)^, where P (*Population*) represents the phenomenon of breastfeeding; C (*Concept*), the Interactive Theory of Breastfeeding; C (*Context*), Nursing clinical practice. Thus, the following question was defined: How can the Interactive Theory of Breastfeeding contribute to Nursing clinical practice?

The search was carried out in the databases Latin American and Caribbean Literature in Health Sciences (LILACS), Specialized Bibliographic Database in the area of Nursing (BDENF), Scientific Electronic Library Online (SCIELO), and Medical Literature Analysis and Retrieval System Online (MEDLINE). The multilingual thesaurus Health Sciences Descriptors (DeCS) and the Medical Subject Headings (MeSH) were used. The terms applied in the Portuguese and English versions were: *Aleitamento Materno*/Breastfeeding; *Enfermagem*/Nursing e *Teoria de Enfermagem*/Nursing Theory*.* The term *Teoria Interativa de Amamentação*/Interactive Theory of Breastfeeding was also used in the search strategy. As a strategy, possible combinations with at least two of these terms were used, using the Boolean operator “AND”. In all strategies the DeCS/MeSH *Teoria de Enfermagem/*Nursing Theory or the term *Teoria Interativa de Amamentação*/Interactive Theory of Breastfeeding were present.

The search was carried out by accessing the Journal Portal of the Coordination for the Improvement of Higher Education Personnel (CAPES). In addition to the aforementioned databases, the Brazilian Digital Library of Theses and Dissertations was also accessed. In the initial search, 55 studies were selected, according to the inclusion criteria: studies available in the data sources that used the Interactive Theory of Breastfeeding in clinical nursing practice, implementation of protocols, and/or nursing interventions. Of the 55 studies, seven were excluded because they were not available in full. The articles were then deposited in the *Rayyan software,* where the 21 duplicates were removed, resulting in a sample of 27 remaining articles. The titles and abstracts were analyzed by two independent reviewers, and four more studies were excluded. The remaining 23 were read in full by both reviewers. At the end of the analysis, 12 studies were excluded because they did not answer the guiding question. Thus, 11 articles were included, in addition to one resulting from the reverse search. The total sample consisted of 12 productions related to the use of the Interactive Theory of Breastfeeding. It should be noted that there was no disagreement among the reviewers.

Data were extracted and deposited on a table as part of the data extraction and analysis process.

For the fourth question, consultations were carried out in Legislation/Resolutions that support the professional practice of nursing. For the fifth question, which assesses the compatibility and expectations of the Interactive Theory of Breastfeeding and nursing practice, it was necessary to know in which context it can be applied.

## RESULTS

In response to the first question, as the theory is based on the breastfeeding process and the skills required for its implementation, it is expected that the application of this practice will be mastered, involving advice and specific techniques, with empathy and without judgment. Many of these skills are included in their Nursing training, which facilitates the theory application.

However, some more peculiar aspects require a certain amount of practice acquired through specific training, such as training as lactation consultants. Therefore, it is important to emphasize that even if Nurses have such skills resulting from their training, clinical management skills in breastfeeding are necessary.

For the second question, it was seen that the theory has been applied in an ascending manner. The theory and the Interactive Breastfeeding Scale are applied daily as a theoretical-practical framework in the breastfeeding extension project, in research and in undergraduate teaching.

The Interactive Theory of Breastfeeding was a theoretical framework for the development of several educational technologies: cartoon about breastfeeding^1*^, application *CareTechAmamenta* ^2**^, serial album on breastfeeding, folders, and instruments for nursing consultation at the Milk Bank or outpatient clinic.

The theory was also used as a theoretical framework for structuring and discussing qualitative research with women breastfeeding after mammoplasty, understanding women’s perceptions of breastfeeding spaces and for describing and interpreting the lactating woman’s comprehension of body image during the breastfeeding period. These studies show women’s realities and experiences in different aspects of their lives and in different social contexts^(13,14,15,16)^

These studies allowed us to evaluate the concepts of the theory, considering that the use of propositions, which are relationships and statements of a middle-range nursing theory on interactive breastfeeding, seemed to be useful to better interpret the subjective experiences of women and support extrapolations that can contribute to the advancement of knowledge on this topic^(4,15)^.

The application of the Interactive Theory of Breastfeeding in these studies was based on three justifications: greater approximation of middle-range theories with clinical practice when compared to grand theories or conceptual models; breastfeeding as the main approach of the theory; and the interactionist basis, which interrelates explicitly personal and social systems^(6)^.

Furthermore, they address elements that help describe the breastfeeding experience of lactating women in the contexts investigated (women who have undergone mammoplasty; women breastfeeding in public spaces; and women’s perception of their body image during breastfeeding), including: biological conditions, the space for breastfeeding, body image, the role of the mother, organizational systems of protection, promotion and support, and women’s decision-making^(13,14,15)^.

Fawcett’s third question analyzes the feasibility of implementing clinical protocols derived from the theory, highlighting several aspects that can be measured and evaluated through specific instruments, in addition to the theory describing, explaining, predicting, and prescribing breastfeeding as a phenomenon^(4,5)^. This reflects on the viability of clinical application, especially built from the theoretical propositions of the Interactive Theory of Breastfeeding, the terminological subset of the International Classification for Nursing Practice (ICNP®) for assistance to women, children, and families in the breastfeeding process, and the results of the Interactive Breastfeeding Scale (EINA).

The ICNP® terminology subset for assistance to women, children, and families in the breastfeeding process consisted of 50 diagnoses/outcomes and 350 nursing interventions, structured and organized by the Interactive Theory of Breastfeeding. Nursing diagnoses, outcomes, and interventions obtained content validity indexes considered capable of being applied to clinical practice during nursing care for women, children, and families in the context of breastfeeding^(17,18)^.

The subset validation process involved nurses from all regions of Brazil, which highlights the representativeness of the ICNP® statements in clinical practice experienced in maternity wards, Neonatal Intensive Care Units, Human Milk Banks, and in primary care^(19)^.

This subset has been incorporated into the application *CareTechAmamenta* and is available in the PlayStore for Androids. The subset has been used in the development of the nursing process with students of the course of Attention to the Health of Women, Children, and Adolescents of the Nursing undergraduate course at Universidade Federal do Espírito Santo and by students of the AMAMENTA extension project, since it helps the student in identifying factors that positively or negatively influence women in the breastfeeding process, in critical thinking and decision-making and, in turn, in the selection of nursing diagnoses/outcomes and interventions.

A protocol was also developed for assisting women during the lactation process, containing diagnoses/outcomes and nursing interventions^(19,20)^. The protocol consists of seven diagnoses/outcomes and 86 nursing interventions based on ICNP^®^ and guided by the Interactive Theory of Breastfeeding for the application of the nursing process centered on the lactating woman, aiming to prevent initial difficulties in the lactation process and improve breastfeeding rates.

The protocol of diagnoses, results and interventions presents a broad scope of the role of nursing in assisting the lactation period, being consistent with the integral and interactive vision of the Interactive Theory of Breastfeeding, and the nurse has multidimensions for his/her action^(20)^.

Regarding the fourth question, it is considered that the theory is based on several concepts and constructs related to the discipline and practice of nursing; therefore, this ensures that the clinician will have the legal support to develop a practice supported by the Interactive Theory of Breastfeeding in conditions of interactive breastfeeding. The use of the subset and the Scale can help to verify the effectiveness of nursing actions.

Regarding the legal capacity of the Nurse, their actions are based on the Code of Ethics, in accordance with the Fundamental Principles, to act in health promotion, prevention, recovery, and rehabilitation, with autonomy and following ethical and legal precepts, which aim to satisfy the health needs of an individual or a population^(21)^. This document is a normative instrument that directs the practice of Nursing in its triad of action (care, teaching, and research), based on guiding principles.

In its latest update, in 2017, it emphasizes its role in health education as an important guide for planning imminently decisive interventions for health promotion, contributing to care planning. In addition to this document and many others, there is Resolution No. 672/2021 which regulates the role and responsibility of Nurses in assisting postpartum women, including assistance with breastfeeding^(22)^.

For the fifth question, it can be seen that Nursing acts autonomously and based on Evidence, mainly associating its main findings with Nursing theories and thus, having good recognition by society in general. This way, their actions are consistent with their expectations.

The use of standardized language in nursing, such as the ICNP^®^ taxonomy, is a good example of a technological tool that contributes to the autonomy and visibility of nurses in their practice.

The ICNP^®^ terminology subset for assistance to women and children in the breastfeeding process was developed through a cross-sectional study, with data collected through systematic and non-participatory observation, during care for postpartum women in a maternity hospital. Postpartum women and their newborns, nurses, and nursing technicians participated. An instrument was used with the 213 interventions of the subset^(17,23)^.

As a result of the 15 observations, 24 interventions were prescribed and observed, such as examining the mother’s breasts; 77 were not prescribed and observed, such as encouraging breastfeeding on free demand; and 112 were not followed or prescribed, such as emphasizing the advantages of breastfeeding. This study concluded that the interventions of the ICNP^®^ subset are applicable in rooming-in. Deficiencies were identified in the prescription and evaluation of nursing interventions^(17,23)^.

To describe the construction and content validation of operational definitions and statements of nursing diagnoses, outcomes, and interventions contained in the ICNP Subset^®^ to assist women, children, and families in the breastfeeding process, a methodological study was carried out. As a result, it was found that of the 58 operational definitions, 54 were validated (93.1%), 39 with a Concordance Index ≥0.8 (67.2%) and 15 (25.8%) with a Concordance Index between ≥0.70 and <0.80. Fifty-four operational definitions, 6 nursing diagnoses/outcomes, and 29 nursing interventions were validated to compose the ICNP^®^ Terminology Subset to assist with the breastfeeding process^(18,19)^.

This subset was developed to guide the practice of nurses who assist women, children, and families in the breastfeeding process, based on a comprehensive and systemic theoretical construction, with the aim of contributing to the standardized nursing language^(17)^.

For the sixth question, the answer is yes. These nursing actions must be based on accurate diagnoses to be chosen appropriately so as to lead to favorable outcomes. Thus, achieving favorable outcomes is a consequence of good investigation of clinical indicators and determination of the best interventions.

In this regard, research was developed to infer the diagnosis of ineffective breastfeeding. The following clinical indicators were shown to be relevant: discontinuity of breast suction; inability of the infant to latch the nipple-areola region correctly; occurrence of the infant crying in the first hour after breastfeeding, and perceived inadequate milk supply^(24)^.

These indicators are present for evaluation based on EINA sentences, so that the application of the Scale contributes to the identification of the most relevant indicators in the evaluation of the nursing diagnosis of ineffective breastfeeding, and in the selection of interventions based on the ICNP^®^ subset and in the evaluation of the results achieved, after new application of the EINA^(5,25,26)^.

Based on the findings of this research and the organization of the concepts of the Interactive Theory of Breastfeeding and aiming at interactive and effective breastfeeding, it is suggested that nurses who assist the mother-child binomial during the breastfeeding process use nursing taxonomies with a focus on accurate inference of diagnoses, directing targeted interventions and evaluating the outcomes obtained.

**Figure 2:**
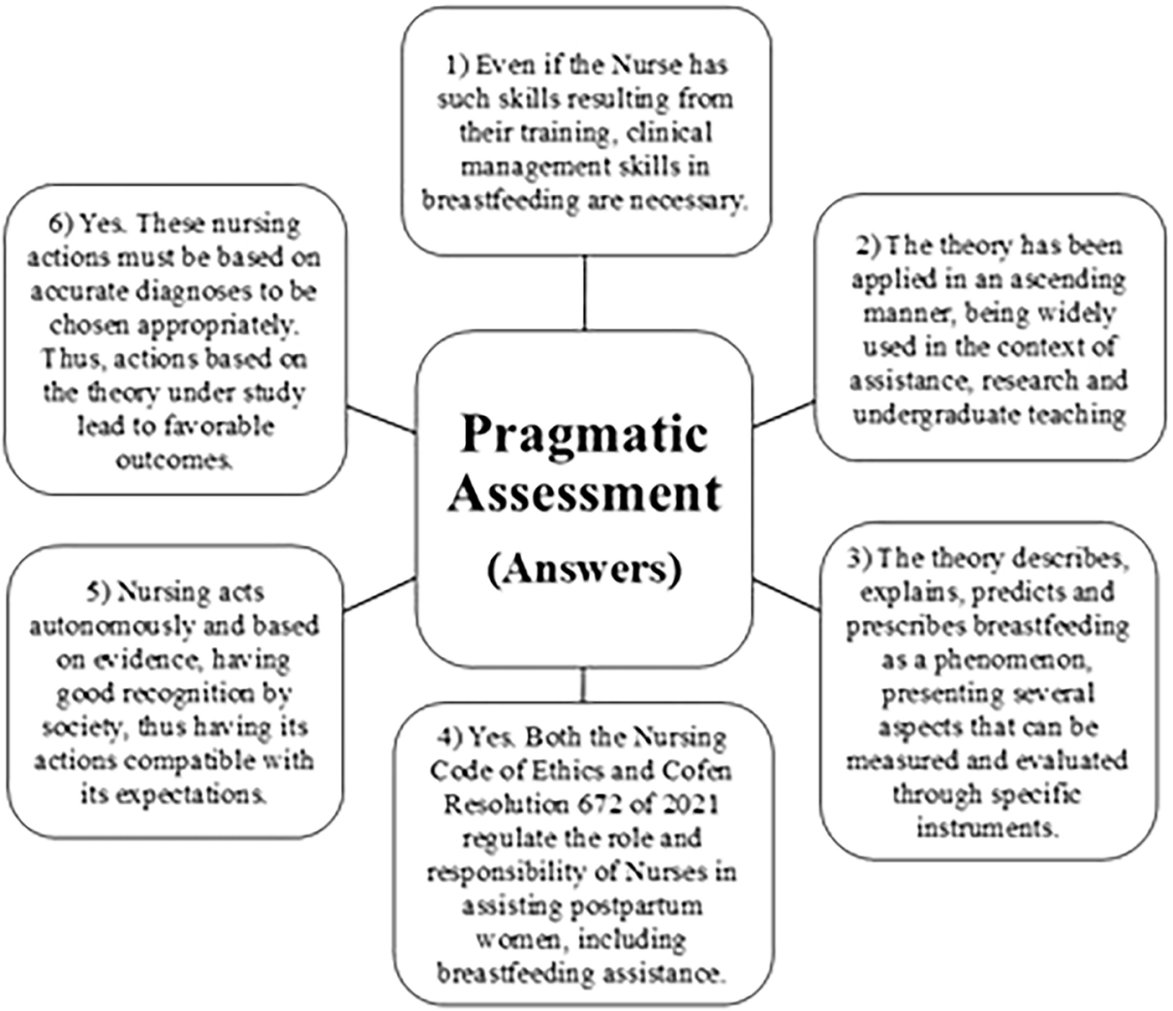
Responses to the pragmatic evaluation of the Interactive Theory of Breastfeeding.

Source: Authors’ production

Thus, we have the synthesis of answers to the questions proposed by Fawcett, described in Figure 2.

## DISCUSSION

The basic purposes of the proposed theory are to describe and explain the phenomenon by analyzing the factors that precede and influence the breastfeeding process^(4)^. To achieve this, skills training is necessary, which involves counseling, specific techniques for managing breastfeeding with empathy and without judgment^(27)^.

Professional training in breastfeeding regarding knowledge and skills is of utmost importance for the perception of breastfeeding as an interactive process, so that the professional can identify weaknesses in the interaction, outlining effective strategies that can ensure adequate guidance to these mothers with the aim of improving their performance during the breastfeeding period.

Therefore, as the nurse is a professional who works to assist women during their breastfeeding process, and has the role of educating these women and their support network, it is important for them to improve their clinical knowledge about lactation^(22)^. Understanding the physiology of this process also becomes essential for the nurse’s performance^(13,27)^.

It is also important to highlight that the Professional Practice Law (LEP) no. 7.498/86 and Decree no. 94.406/87, which would correspond to the “Magna Carta” for the nursing practice, define the competences, duties, and obligations of nursing professionals, specifying each level of responsibility^(28,29)^.

In this sense, Nursing, which already has its practice assured by LEP, has been increasingly improving its knowledge in the application of the exercise of care, teaching and research, and, currently, nurses are committed to discussing their clinical role in Nursing^(30)^.

Furthermore, understanding the multi-dimensions involved in the conceptual structure of the Interactive Theory of Breastfeeding is essential for the behaviors that provide different types of benefits from breastfeeding and its dynamic mother-child interaction. These theoretical concepts cover aspects that can help nurses apply the theory, which are: dynamic mother-child interaction; women’s biological conditions; children’s biological conditions; women’s perception; children’s perception; women’s body image; space for breastfeeding; role of mother; organizational systems for the protection, promotion, and support of breastfeeding; family and social authority; women’s decision-making; stress and breastfeeding time^(4,5)^.

This theory can be used in care actions using the Interactive Breastfeeding Scale (EINA). In the last two decades, scales or tools have been developed to measure aspects that influence breastfeeding, such as knowledge, behaviors, attitudes and biological-psychosocial variables. This strengthens the support, promotion and protection of breastfeeding with a view to improving maternal and child health^(5)^.

There are several worldwide Scales related to breastfeeding. In Brazil, the most used is the Canadian Breastfeeding Self-Efficacy Scale *–* BSES, adapted to the country’s cultural reality, acting only in relation to women’s reliability. The Interactive Breastfeeding Scale, in its turn, is capable of guiding clinical practice to a greater extent^(5,25)^.

The theory under analysis proposes work based on certain theoretical concepts related to interaction and breastfeeding. Encouraging the construction of bonds and interaction between mother and child, and promoting correct latch adjustment, for example, are nursing actions provided for by Resolution No. 672/2021^(22)^.

The nurse supports and promotes breastfeeding, identifying and intervening in difficulties from the gestational period to the postpartum period. This care is essential to prevent early weaning^(16,30)^. Furthermore, they are capable of recognizing factors that interfere in the dynamics of breastfeeding, allowing comprehensive and interactive interaction with the binomial and inferring nursing diagnoses^(4,5)^.

The protection, promotion, and support of the breastfeeding process consider the respect for maternal conditions in relation to their physiology, as well as understand women and children’s behavior through humanized and empathetic assistance^(12,15)^.

In the nursing clinical practice, the use of Interactive Theory of Breastfeeding offers significant contributions by fostering understanding and promoting successful breastfeeding interactions. The theory emphasizes the dynamic and systemic aspects of breastfeeding, analyzing factors that influence the process and its results. Moreover, the development of tools, such as the Interactive Breastfeeding Scale, helps to assess the interaction between mother, child, and environment during breastfeeding, guiding clinical practice and helping professionals to assess the factors that impact this dynamic interaction.

As limitations of the study, there is the recent appearance of the Theory, with few records of its applicability, as well as a greater number of national publications, which hinders the dissemination of knowledge in databases focusing on international publications.

## CONCLUSION

Fawcett’s pragmatic adequacy was achieved by the theory under study, as it was able to fully understand the breastfeeding process, helping nurses to evaluate the various factors that interfere in this phenomenon.

The pragmatic adequacy criterion requires these professionals to undergo training in skills in breastfeeding management, as well as in the application of the theory in question, which was developed in clinical practice to assist in research, teaching and nursing care, bringing scientific rigor to their work. The Interactive Theory of Breastfeeding is useful for maternal and child clinical practice, being able to help nurses, as well as other professionals, to achieve knowledge, skills, and decision-making abilities to promote breastfeeding.
