# Supplementary material for: Interactive breastfeeding theory: fawcettʼs pragmatic adequacy assessment
Source: Rev Esc Enferm USP. 2024 Dec 2;58:e20240144. doi: 10.1590/1980-220X-REEUSP-2024-0144en (PMC11613921; doi:10.1590/1980-220X-REEUSP-2024-0144en)
Supplement: Translation

# Translation: Teoria interativa de amamentação: avaliação da adequação pragmática de fawcett

**DOI:** 10.1590/1980-220X-REEUSP-2024-0144pt

**Authors:** Samara Calixto Gomes, Icleia Parente Rodrigues, Marcos Venícius de Oliveira Lopes, Viviane Martins da Silva, Nirla Gomes Guedes, Patricia Neyva da Costa Pinheiro

**Affiliations:** 1 Universidade Federal do Ceará, Departamento de Enfermagem, Fortaleza, CE, Brasil.

**Keywords:** Teoria de enfermagem, Estudo de avaliação, Saúde materno-infantil, Aleitamento materno

## Abstract

**Objetivo:**

Realizar a avaliação da adequação pragmática da Teoria Interativa de Amamentação a partir do Modelo de Fawcett.

**Método:**

Estudo teórico, conforme critérios de avaliação proposto por Fawcett. Utilizaram-se as seis perguntas sugeridas no modelo, respondidas a partir da busca na literatura científica, consulta em legislações que respaldam o exercício profissional da enfermagem e informações advindas dos autores da teoria.

**Resultados:**

Observou-se que durante a sua formação, o Enfermeiro adquire habilidades para o cuidado, mas é necessário intensificar o manejo clínico voltado à amamentação. Desde sua criação, a teoria é aplicada de forma ascendente, mostrando-se viável a sua aplicação na prática clínica. A existência de leis e resoluções garantem ao enfermeiro clínico o amparo legal necessário para o desenvolvimento de uma prática suportada na Teoria Interativa de Amamentação.

**Conclusão:**

A adequação pragmática da teoria foi observada, mostrando que a Teoria Interativa de Amamentação subsidia um cuidado direcionado ao binômio mãe e filho, dando suporte ao enfermeiro em suas tomadas de decisões, contribuindo para a proteção, promoção e apoio à amamentação.

## INTRODUÇÃO

Uma pesquisa realizada sobre amamentação em 153 países destaca o Brasil como uma referência mundial, registrando uma taxa de 41% de adesão^(1)^. Ainda assim, há muito o que caminhar para alcançarmos os 70% definidos como meta até 2030, pela Organização Mundial da Saúde (OMS) e o Fundo das Nações Unidas para a Infância (UNICEF)^(2)^.

O ato de amamentar é muito mais que uma simples oferta de leite humano. É uma prática multifatorial e complexa que pode ser influenciada diretamente por alguns aspectos, tais como: a percepção e condições biológicas do binômio mãe e filho, espaço para amamentar, papel de mãe e sua tomada de decisão^(3)^.

Na complexidade que envolve a amamentação, destaca-se a Teoria Interativa de Amamentação que a descreve como uma dimensão sistêmica, dinâmica e processual. É considerada “um processo de interação dinâmica no qual mãe e filho interagem entre si e com o ambiente para alcançar os benefícios do leite humano oferecido direto da mama para a criança, sendo uma experiência única a cada evento” ^(3,4)^. Esta teoria apresenta como proposta de referencial teórico o Modelo Conceitual de Sistemas Abertos de Imogene King^(5)^.

No contexto do cuidado na enfermagem, as teorias contribuem como referencial teórico para uma representação simbólica de aspectos da realidade, legitimando uma profissão baseando-se na sua habilidade de criar e aplicar teorias. Na prática, promovem o conhecimento como um guia das ações do enfermeiro^(6)^.

As teorias apresentam elementos capazes de descrever, explicar, prever ou prescrever condições ou relações entre fenômenos^(5,7)^. Ao aplicá-las, a enfermagem promove um meio sistemático para a realização de uma reflexão crítica, auxiliando o exercício do enfermeiro baseados em referenciais teóricos para sua prática^(8)^.

Uma teoria geralmente surge da necessidade de expressar uma nova ideia ou fenômeno, sendo um importante elo entre conhecimento e prática^(6)^. O uso de teorias estrutura e organiza conhecimentos de enfermagem, proporcionando autonomia na assistência, trazendo reflexões importantes para a visibilidade da enfermagem como ciência^(8)^.

Assim, vale ressaltar a importância de avaliar uma teoria e seus componentes para o aprimoramento do desenvolvimento da prática do enfermeiro, sendo capaz de direcionar a estruturação de pesquisas, ensino, administração e a consulta de enfermagem, efetivando mudanças na prática clínica e incentivando o enfermeiro a ser consumidor crítico de teorias e da prática baseada em evidências^(7)^.

Para ser avaliada, uma teoria requer que sejam feitos julgamentos sobre até que ponto esta satisfaz certos critérios. Assim, Fawcett^(9)^ determina, como um de seus critérios de avaliação de uma teoria, a Adequação Pragmática, que se refere à utilidade de uma teoria na prática de enfermagem e a medição dos resultados em termos da eficácia na resolução de problemas.

Dessa forma, este estudo objetiva realizar a avaliação da adequação pragmática da Teoria Interativa de Amamentação a partir do Modelo de Fawcett. O estudo dessa teoria justifica- se com o escopo de aprofundar a compreensão e difundir seus conceitos para a prática clínica do enfermeiro.

## MÉTODO

Estudo teórico de avaliação da aplicabilidade prática da Teoria Interativa de Amamentação, utilizando os critérios de avaliação propostos por Jacqueline Fawcett^(9)^, que envolvem julgamentos relacionados à confiabilidade de uma teoria, definidos em seis critérios: significância, consistência interna, parcimônia, testabilidade, adequação empírica e, por último, a adequação pragmática^(10)^.

A adequação pragmática é o sexto critério de avaliação de uma teoria e baseia-se na sua aplicabilidade prática e exige dos enfermeiros entendimento sobre seu conteúdo e habilidades necessárias para a sua aplicação, para que possam alcançar resultados favoráveis das ações de cuidado^(9)^. Essa abordagem enfatiza a eficácia de uma teoria na solução de problemas e procura determinar se o que é pretendido ou experimentado atinge seu propósito^(10)^.

Esse critério requer que a partir de ações significativas, a teoria promova resultados positivos, como: redução das complicações, melhoria nas condições de saúde e aumento da satisfação de todos os participantes^(10)^.

Para avaliar a adequação pragmática na Teoria Interativa de Amamentação foram aplicadas as perguntas sugeridas por Fawcett, apresentadas na Figura 1.

**Figura 1:** Perguntas propostas por Fawcett para avaliação pragmática.

Fonte: Produção dos autores

O primeiro questionamento investiga se há a necessidade de um treinamento específico para a aplicação da teoria na prática clínica. Inicialmente, destaca-se o termo competências como a “capacidade de articular valores, conhecimentos, habilidades e atitudes necessários para o desempenho eficaz de atividades requeridas pela natureza do trabalho, além do alcance dos objetivos estabelecidos”^(11)^. Neste sentido, os termos: *Skills* e *Soft Skills* devem ser reconhecidos, onde o primeiro refere-se às competências técnicas, que podem ser validadas de forma direta, por meio de testes objetivos, ou indiretamente, pela apresentação de um certificado de conclusão de algum curso e, este último, refere-se às competências socioemocionais, ou não técnicas, como capacidade de comunicação, habilidade de trabalho em equipe e liderança^(11)^.

Para mapear as respostas da segunda, terceira e sexta questões, realizou-se uma revisão de escopo baseada nas recomendações do JBI^(12)^, seguindo as seguintes etapas: definição e alinhamento dos objetivos e pergunta norteadora; desenvolvimento dos critérios de inclusão e exclusão; descrição do planejamento de busca, seleção, extração de dados e apresentação de resultados.

O objetivo da revisão foi mapear as contribuições da Teoria Interativa de Amamentação para a prática clínica da enfermagem. A pergunta de pesquisa foi formulada com base na estratégia PCC, que corresponde ao acrônimo *Population, Concept, Context* (PCC)^(12)^, onde P (*Population*) representa o fenômeno da amamentação; C (*Concept*), a Teoria Interativa de Amamentação; C (*Context*), a prática clínica da Enfermagem. Assim, definiu-se a seguinte pergunta: Como a Teoria Interativa de Amamentação pode contribuir para a prática clínica da Enfermagem?

A busca foi realizada nas bases de dados Literatura Latino-Americana e do Caribe em Ciências da Saúde (LILACS), Base de Dados Bibliográficas Especializada na área de Enfermagem (BDENF), Scientific Electronic Library Online (SCIELO) e *Medical Literature Analysis and Retrieval System Online* (MEDLINE). Foram utilizados o tesauro multilíngue Descritores em Ciências da Saúde (DeCS) e os *Medical Subject Headings* (MeSH). Os termos aplicados na versão português e inglês foram: Aleitamento Materno/*Breastfeeding*; Enfermagem/Nursing e Teoria de Enfermagem/*Nursing Theory.* Na estratégia de busca também foi utilizado o termo Teoria Interativa de Amamentação/*Interactive Theory of Breastfeeding*. Como estratégia foram utilizadas as combinações possíveis com no mínimo dois desses termos, utilizando o operador booleano “AND”. Em todas as estratégias o DeCS/MeSH Teoria de Enfermagem/*Nursing Theory* ou o termo Teoria Interativa de Amamentação/*Interactive Theory of Breastfeeding* estavam presentes.

Procedeu-se a busca a partir do acesso ao Portal de Periódicos da Coordenação de Aperfeiçoamento de Pessoal de Nível Superior (CAPES). Além das bases acima referidas, procedeu-se com o acesso à Biblioteca Digital Brasileira de Teses e Dissertações. Na busca inicial foram selecionados 55 estudos, de acordo com os critérios de inclusão: estudos disponíveis nas fontes de dados que utilizaram a Teoria Interativa de Amamentação na prática clínica de enfermagem, implementação de protocolos e /ou intervenções de enfermagem. Dos 55 estudos, sete foram excluídos por não estarem disponíveis na íntegra. Em seguida, os artigos foram depositados no *software Rayyan,* onde os 21 duplicados foram removidos, contemplando uma amostra de 27 restantes. Os títulos e resumos foram analisados por dois revisores independentes, sendo excluídos mais quatro estudos. Os 23 restantes foram lidos na íntegra pelos dois revisores. Ao final da análise, foram excluídos 12 estudos por não responderam à questão norteadora. Desse modo, foram incluídos 11 artigos, além de um advindo da busca reversa. O total da amostra foi de 12 produções relacionadas ao uso da Teoria Interativa de Amamentação. Ressalta-se que não houve divergência entre os revisores.

Os dados foram extraídos e depositados em uma tabela como parte do processo de extração e análise dos dados.

Para a quarta questão foram realizadas consultas em Legislações/Resoluções que respaldam o exercício profissional da enfermagem. E para o quinto questionamento, que avalia a compatibilidade e expectativas da Teoria Interativa de Amamentação e a prática de enfermagem, fez-se necessário conhecer em qual contexto ela pode ser aplicada.

## RESULTADOS

Em resposta ao primeiro questionamento, como a teoria é baseada no processo de amamentação e as habilidades necessárias para sua implementação, prevê-se o domínio acerca da aplicação dessa prática que envolve aconselhamento e técnica específica, com empatia e sem julgamentos. Grande parte dessas competências estão inclusas na sua formação em Enfermagem, o que facilita a aplicação dessa teoria.

Entretanto, alguns aspectos mais peculiares requerem uma certa prática adquirida por meio de treinamentos específicos, como a formação de consultores em lactação. Assim, é importante salientar que mesmo o Enfermeiro possuindo tais habilidades advindas de sua formação, é necessária uma habilidade de manejo clínico em amamentação.

Para a segunda pergunta, viu-se que a teoria vem sendo aplicada de forma ascendente. Diariamente a teoria e a Escala Interativa de Amamentação são aplicadas como arcabouço teórico-prático no projeto de extensão Amamenta, em pesquisas e no ensino da graduação.

A Teoria Interativa de Amamentação foi marco teórico para o desenvolvimento de várias tecnologias educacionais: desenho animado sobre amamentação^1*^, aplicativo *CuidarTechAmamenta* ^2**^, álbum seriado sobre amamentação, folders, e instrumentos para consulta de enfermagem em Banco de Leite ou ambulatório.

A teoria também foi utilizada como referencial teórico para estruturação e discussão de pesquisas qualitativas com mulheres em amamentação após mamoplastia; para conhecer a percepção da mulher sobre os espaços para amamentar e para descrever e interpretar a compreensão da lactante acerca da imagem corporal no período da amamentação. Essas pesquisas trazem as realidades e vivências das mulheres em diferentes aspectos de suas vidas e contextos sociais^(13,14,15,16)^.

Estas pesquisas permitiram avaliar os conceitos da teoria, tendo em vista que o uso de proposições, que são relações e afirmativas de uma teoria de enfermagem de médio alcance sobre a amamentação interativa, pareceu ser útil para melhor interpretar as experiências subjetivas das mulheres e sustentar extrapolações que possam contribuir no avanço do conhecimento desta temática^(4,15)^.

A aplicação da Teoria Interativa de Amamentação nessas pesquisas ancorou-se em três justificativas: maior aproximação das teorias de médio alcance com a prática clínica quando comparadas às grandes teorias ou modelos conceituais; a amamentação como abordagem principal da teoria; e a base interacionista, que interrelaciona de maneira explícita sistemas pessoais e sociais^(6)^.

Ainda mais, abordam elementos que auxiliam na descrição da experiência da amamentação das lactantes nos contextos investigados (mulheres submetidas a mamoplastia; mulheres amamentando em espaços públicos; e a percepção das mulheres acerca da sua imagem corporal durante a amamentação), dentre eles: as condições biológicas, o espaço para amamentar, a imagem corporal, o papel da mãe, os sistemas organizacionais de proteção, promoção e apoio e tomada de decisão da mulher^(13,14,15)^.

O terceiro questionamento de Fawcett analisa a viabilidade da implementação de protocolos clínicos derivados da teoria, destacando-se diversos aspectos que podem ser mensurados e avaliados por meio de instrumentos específicos, além de a teoria descrever, explicar, predizer e prescrever a amamentação como fenômeno^(4,5)^. Isso reflete na viabilidade da aplicação clínica, especialmente construída a partir das proposições teóricas da Teoria Interativa de Amamentação, do subconjunto terminológico da Classificação Internacional para a Prática de Enfermagem (CIPE^®^) para assistência à mulher, à criança e à família em processo de amamentação e dos resultados da Escala Interativa de Amamentação (EINA).

O subconjunto terminológico da CIPE^®^ para assistência à mulher, à criança e à família em processo de amamentação foi composto por 50 diagnósticos/resultados e 350 intervenções de enfermagem, estruturado e organizado pela Teoria Interativa de Amamentação. Os diagnósticos, resultados e intervenções de enfermagem obtiveram índices de validade de conteúdo considerados capazes de serem aplicáveis à prática clínica durante assistência de enfermagem à mulher, à criança e à família no contexto da amamentação^(17,18)^.

O processo de validação do subconjunto envolveu enfermeiros de todas as regiões do Brasil, o que evidencia a representatividade dos enunciados CIPE^®^ na prática clínica vivenciados em maternidade, Unidade de Terapia Intensiva Neonatal, Banco de Leite Humano e na atenção primária^(19)^.

Esse subconjunto foi incorporado ao aplicativo *CuidarTechAmamenta* e está disponível na loja *PlayStore* para *Androids*. O subconjunto vem sendo utilizado no desenvolvimento do processo de enfermagem com os acadêmicos da disciplina de Atenção à Saúde da Mulher, Criança e Adolescente do Curso de graduação em Enfermagem da Universidade Federal do Espírito Santo e pelos estudantes do projeto de extensão AMAMENTA, uma vez que auxilia o estudante na identificação de fatores que influenciam positivamente ou negativamente a mulher em processo de amamentação, no pensamento crítico e na tomada de decisões e, por sua vez, na seleção dos diagnósticos/resultados e intervenções de enfermagem.

Também foi desenvolvido um protocolo para a assistência à mulher em processo de lactação contendo diagnósticos/resultados e intervenções de enfermagem^(19,20)^. O protocolo consta de sete diagnósticos/resultados e 86 intervenções de enfermagem baseados na CIPE^®^ e orientados pela Teoria Interativa de Amamentação para a aplicação do processo de enfermagem centrado na lactante, tendo em vista a prevenção das dificuldades iniciais do processo de lactação e melhora das taxas de amamentação.

O protocolo de diagnósticos, resultados e intervenções apresenta um amplo alcance do papel da enfermagem assistindo o período da lactação, sendo compatível com a visão integral e interativa da Teoria Interativa de Amamentação, e o enfermeiro tem multidimensões para o seu agir^(20)^.

Em relação à quarta pergunta, considera-se que a teoria é baseada em diversos conceitos e construtos relacionados à disciplina e prática da enfermagem; então, isto garante ao clínico o amparo legal de desenvolver uma prática suportada na Teoria Interativa de Amamentação em condições da amamentação interativa. O uso do subconjunto e da Escala pode ajudar a verificar a eficácia das ações de enfermagem.

Quanto à capacidade legal do Enfermeiro, suas ações são baseadas no Código de Ética, de acordo com os Princípios Fundamentais, para atuar na promoção, prevenção, recuperação e reabilitação da saúde, com autonomia e em consonância com os preceitos éticos e legais, que visem satisfazer as necessidades de saúde de um indivíduo ou uma população^(21)^. Tal documento é um instrumento normativo que direciona a prática da Enfermagem na sua tríade de atuação (assistência, ensino e pesquisa), baseando-se em princípios norteadores.

Em sua última atualização, em 2017, enfatiza a sua atuação na educação em saúde como importante direcionador para planejamento de intervenções iminentemente decisivas para a promoção da saúde, contribuindo para o planejamento do cuidado. Além desse documento e tantos outros, há a Resolução nº 672/2021 que normatiza a atuação e a responsabilidade do Enfermeiro à assistência a Puérperas, incluindo assistência ao aleitamento materno^(22)^.

Para o quinto questionamento, vê-se que a Enfermagem atua de forma autônoma e baseada em Evidências, principalmente associando seus principais achados às teorias de Enfermagem e assim, tendo um bom reconhecimento pela sociedade em geral. Dessa forma, suas ações são compatíveis a suas expectativas.

O uso de linguagem padronizada na enfermagem, como a taxonomia CIPE^®^, é um bom exemplo de um instrumental tecnológico que contribui para a autonomia e visibilidade do enfermeiro na sua prática.

O subconjunto terminológico da CIPE^®^ para assistência à mulher e à criança em processo de amamentação foi desenvolvido através de um estudo transversal, com dados coletados pela observação, sistemática e não participativa, durante os cuidados às puérperas em uma maternidade. Participaram puérperas e seus recém-nascidos, enfermeiras e técnicos em enfermagem. Foi utilizado instrumento com as 213 intervenções do subconjunto^(17,23)^.

Como resultados das 15 observações, 24 intervenções foram prescritas e observadas, como examinar as mamas da mãe; 77 não prescritas e observadas, como estimular amamentação em livre demanda; e 112 não foram observadas e nem prescritas, como reforçar as vantagens da amamentação. Este estudo concluiu que as intervenções do subconjunto da CIPE^®^ são aplicáveis em alojamento conjunto. Identificou-se deficiência na prescrição e avaliação das intervenções de enfermagem^(17,23)^.

Para descrever a construção e validação de conteúdo de definições operacionais e de enunciados de diagnósticos, resultados e intervenções de enfermagem contidos no Subconjunto da CIPE^®^ para assistência à mulher, à criança e à família em processo de amamentação foi realizado um estudo metodológico. Como resultado, encontrou-se que das 58 definições operacionais, 54 foram validadas (93,1%), sendo 39 com Índice de Concordância ≥0,8 (67,2%) e 15 (25,8%) com Índice de Concordância entre ≥0,70 e <0,80. Foram validadas 54 definições operacionais, 6 diagnósticos/resultados de enfermagem e 29 intervenções de enfermagem para compor o Subconjunto Terminológico da CIPE^®^ para assistência ao processo de amamentação^(18,19)^.

Esse subconjunto foi desenvolvido para orientar a prática dos enfermeiros que assistem mulheres, crianças e famílias em processo de amamentação, tendo por base uma construção teórica abrangente e sistêmica, com objetivo de contribuir na linguagem padronizada de enfermagem^(17)^.

Para o sexto questionamento, a resposta é sim. Essas ações de enfermagem devem estar baseadas em diagnósticos acurados para serem escolhidas adequadamente de forma a levar a resultados favoráveis. Assim, o alcance de resultados favoráveis são consequência de uma boa investigação de indicadores clínicos e determinação das melhores intervenções.

Nesse sentido, desenvolveu-se uma pesquisa para a inferência do diagnóstico de amamentação ineficaz. Mostraram-se relevantes os seguintes indicadores clínicos: descontinuidade da sucção da mama; incapacidade do lactente de apreender a região areolomamilar corretamente; ocorrência de choro do lactente na primeira hora após a amamentação e suprimento de leite inadequado percebido^(24)^.

Esses indicadores estão presentes para avaliação a partir de sentenças da EINA, de forma que a aplicação da Escala contribui para a identificação dos indicadores mais relevantes na avaliação do diagnóstico de enfermagem de amamentação ineficaz, e na escolha das intervenções a partir do subconjunto CIPE^®^ e na avaliação dos resultados alcançados, após nova aplicação da EINA^(5,25,26)^.

Com base nos achados dessa pesquisa e na organização dos conceitos da Teoria Interativa de Amamentação e no intuito de uma amamentação interativa e eficaz, sugere-se aos enfermeiros que assistem ao binômio mãe-filho durante o processo de amamentação a utilização de taxonomias de enfermagem com foco na inferência acurada de diagnósticos, no direcionamento de intervenções direcionadas e na avaliação dos resultados obtidos.

Assim, tem-se a síntese de respostas às perguntas propostas por Fawcett, descritas na Figura 2.

**Figura 2:** Respostas da avaliação pragmática da Teoria Interativa de Amamentação.

Fonte: Produção dos autores.

## DISCUSSÃO

As finalidades basais da teoria proposta são de descrever e explicar o fenômeno ao analisar os fatores que antecedem e que influenciam o processo de amamentar^(4)^. Para isso, faz-se necessário treino de habilidades que envolve aconselhamento, técnica específica do manejo da amamentação com empatia e sem julgamentos^(27)^.

A capacitação profissional em aleitamento materno sobre os conhecimentos e habilidades é de suma importância para a percepção da amamentação como um processo interativo, de modo que o profissional possa identificar fragilidades da interação, traçando estratégias eficazes que possam assegurar orientações adequadas a essas mães com intuito de melhorar o desempenho destas no período da amamentação.

Assim, sendo o enfermeiro um profissional atuante na assistência à mulher durante seu processo de amamentação, e tendo seu papel de educador dessa mulher e sua rede de apoio, é importante seu aperfeiçoamento no conhecimento clínico sobre a lactação^(22)^. Compreender a fisiologia desse processo também se torna imprescindível para a atuação do enfermeiro^(13,27)^.

Também é importante destacar que a Lei do Exercício Profissional (LEP) nº 7.498/86 e o Decreto no 94.406/87, que corresponderia à “Carta Magna’’ para o exercício do enfermeiro, definem as competências, os deveres e as obrigações dos profissionais de enfermagem, especificando cada nível de responsabilidade^(28,29)^.

Nesse sentido, a Enfermagem, que já tem sua prática assegurada pela LEP, vem aperfeiçoando cada vez mais seus conhecimentos na aplicação do exercício da assistência, ensino e pesquisa, e, hoje, o enfermeiro empenha-se em discutir o seu papel clínico na Enfermagem^(30)^.

Ademais, a compreensão das multidimensões envolvidas na estrutura conceitual da Teoria Interativa de Amamentação é essencial às condutas que proporcionem diferentes tipos de benefícios da amamentação e de sua interação dinâmica mãe-filho. Esses conceitos teóricos abrangem aspectos que podem auxiliar o enfermeiro na aplicabilidade da teoria, que são: interação dinâmica mãe-filho; condições biológicas da mulher; condições biológicas da criança; percepção da mulher; percepção da criança; imagem corporal da mulher; espaço para amamentar; papel de mãe; sistemas organizacionais de proteção, promoção e apoio à amamentação; autoridade familiar e social; tomada de decisão da mulher; estresse e tempo de amamentação^(4,5)^.

Essa teoria pode ser utilizada nas ações de cuidado pela Escala Interativa de Amamentação (EINA). Nas últimas duas décadas, foram desenvolvidas escalas ou ferramentas que mensurem aspectos que influenciam a amamentação, como conhecimentos, comportamentos, atitudes e variáveis biológicas-psicossociais. Isso fortalece o apoio, promoção e proteção do aleitamento materno como vistas a apontar melhorias na saúde materno infantil^(5)^.

Existem diversas Escalas mundiais relacionadas à amamentação. No Brasil, a mais utilizada é a canadense *Breastfeeding Self-Efficacy Scale –* BSES, adaptada à realidade cultural do país, atuando apenas em relação à confiabilidade da mulher. Já a Escala Interativa de Amamentação é capaz de direcionar a prática clínica em uma maior amplitude^(5,25)^.

A teoria em análise propõe um trabalho com base em determinados conceitos teóricos relacionados à interação e amamentação. Estimular a construção de vínculos e interação entre mãe e filho, e promover ajuste de pega correta, por exemplo, são ações de enfermagem previstas pela Resolução nº 672/2021^(22)^.

O enfermeiro apoia e promove a amamentação, identificando e intervindo nas dificuldades desde o período gestacional até o puerpério. Esse cuidado é fundamental à prevenção do desmame precoce^(16,30)^. Além disso, é capaz de reconhecer fatores que interferem na dinâmica da amamentação, possibilitando uma interação abrangente e interativa com o binômio e inferir diagnósticos de enfermagem^(4,5)^.

A proteção, promoção e apoio ao processo de amamentar considera o respeito às condições maternas em relação à sua fisiologia, bem como compreender comportamentos da mulher e da criança por meio de uma assistência humanizada e empática^(12,15)^.

Na prática clínica do enfermeiro, a utilização da Teoria Interativa de Amamentação oferece contribuições significativas ao favorecer a compreensão e a promoção de interações bem-sucedidas na amamentação. A teoria enfatiza os aspectos dinâmicos e sistêmicos da amamentação, analisando fatores que influenciam o processo e seus resultados. Além disso, o desenvolvimento de ferramentas, como a Escala Interativa de Amamentação, auxilia na avaliação da interação entre mãe, filho e meio ambiente durante a amamentação, orientando a prática clínica e auxiliando os profissionais na avaliação dos fatores que impactam essa interação dinâmica.

Como limitações do estudo, dá-se a recente existência da Teoria, com poucos registros de sua aplicabilidade, bem como maior número de publicações nacionais, o que dificulta a disseminação de conhecimento em bases de dados com foco em publicações internacionais.

## CONSIDERAÇÕES FINAIS

A adequação pragmática de Fawcett foi atingida pela teoria em estudo, por ser capaz de compreender a plenitude do processo de amamentação, auxiliando enfermeiros a avaliar os diversos fatores que interferem nesse fenômeno.

O critério de adequação pragmática requer desses profissionais treinamentos de habilidades no manejo da amamentação, bem como na aplicação da teoria em questão, que foi desenvolvida na prática clínica auxiliando na pesquisa, ensino e assistência da enfermagem, trazendo rigor científico na sua atuação. A Teoria Interativa de Amamentação apresenta-se útil para a prática clínica materno-infantil, sendo capaz de auxiliar o enfermeiro, bem como outros profissionais, a alcançar conhecimento, habilidades e tomada de decisões para a promoção da amamentação.
